# Current perspective in acute ischemic stroke: net water uptake and imaging-guided selection as bridges to personalized, tissue-based care

**DOI:** 10.3389/fneur.2025.1741898

**Published:** 2026-01-22

**Authors:** Gabriel Broocks, Rene Werner, Thilo Sentker, Jens Minnerup, Andre Kemmling

**Affiliations:** 1 Department of Neuroradiology, HELIOS Medical Center, University Campus of MSH Medical School Hamburg, Schwerin, Germany; 2 Image Processing and Medical Imaging, Institute for Applied Medical Informatics, Institute for Computational Neuroscience, University Medical Center Hamburg-Eppendorf, Hamburg, Germany; 3 Department of Neurology, University Hospital Schleswig-Holstein, University of Lübeck, Lübeck, Germany; 4 Department of Neuroradiology, University Hospital Marburg, Marburg, Germany

**Keywords:** computed tomography, edema, imaging, stroke, thrombectomy

## Abstract

Acute ischemic stroke diagnosis is shifting to individualized, tissue-based treatment decision-making. Recent randomized trials have expanded thrombectomy to patients with large ischemic lesions, although outcomes vary across studies—with MRI-selected cohorts (e.g., LASTE) positive and CT-only selection (e.g., TESLA) neutral in its primary endpoint (1–4). Neuroprotection remains relevant despite neutral/negative results in ESCAPE-NEXT and CHARM, as ultra-early strategies applied in the FRONTIER trial suggest promise (5–7). Tenecteplase has emerged as a practical, effective thrombolytic alternative to alteplase, and trials are testing adjuvant intra-arterial lysis (e.g., TECNO) (8). Conversely, evidence for thrombectomy in low NIHSS stroke and distal/medium vessel occlusions is not yet available or has been neutral to negative in recent randomized trials, underscoring that treatment selection matters (9, 10). This Perspective comment highlights net water uptake (NWU) as a prototypical CT-based edema biomarker alongside novel imaging techniques that may refine selection–stratifying risk, forecasting complications, and identifying patients likely to benefit from reperfusion or anti-edema strategies. We conclude with an outlook on AI-enabled, CT-first workflows that deliver rapid tissue profiling–penumbral imaging, automated early edema imaging such as NWU, and collaterals–to guide personalized acute stroke therapy.

## Current status of acute ischemic stroke treatment

Significant progress has been made in acute ischemic stroke care over the past few years, particularly in expanding treatment to patients who were previously considered untreatable. Multiple recent trials, including TENSION, have demonstrated that endovascular thrombectomy can benefit patients with large infarct cores, a group that was excluded from early trials (3). Several trials showed improved outcomes with thrombectomy in patients with large-core strokes, and the more recent LASTE trial confirmed a clear benefit even when there was no upper limit on infarct size (2). In LASTE, patients with an ASPECTS 0–5 (>50% with ASPECTS 0–2) who were treated with thrombectomy had significantly better 90-day functional outcomes (generalized OR ~1.63) and reduced mortality compared to medical therapy (2). Notably, LASTE allowed advanced imaging with a majority receiving MRI for patient selection and found benefit across all ASPECTS subgroups. By contrast, the TESLA trial, which relied on noncontrast CT alone to identify large-core strokes (ASPECTS 2–5), did not meet its primary endpoint (4). TESLA showed no statistically significant improvement in functional outcome with thrombectomy (utility-weighted mRS mean 2.93 vs. 2.27, with a posterior probability of superiority 0.96 not reaching the prespecified 0.975 threshold) (4). These differences highlight how patient selection criteria and imaging modalities can influence trial outcomes. While advanced imaging may help identify patients who have salvageable tissue despite a large core visible on CT, carefully designed CT-based strategies are also evolving and may be further refined by quantitative markers such as NWU.

Beyond reperfusion therapy, the field has seen renewed interest in neuroprotective strategies, though results have been mixed. The ESCAPE-NA1 and ESCAPE-NEXT trials of nerinetide (NA-1), a neuroprotective peptide, were overall neutral – there was no difference in 90-day outcomes (mRS 0–2) between the nerinetide and placebo groups among thrombectomy patients (11, 12). Similarly, the phase 3 CHARM trial testing intravenous glibenclamide (a Sur1-Trpm4 channel blocker targeting edema) did not improve functional outcomes in patients with large hemispheric infarctions (6). Despite these setbacks, neuroprotection remains a relevant research avenue (13). The FRONTIER trial, which administered nerinetide in the pre-hospital setting via paramedics, hinted at positive effects when treatment was initiated very early. In FRONTIER, nerinetide given within ~60 min of stroke onset showed a substantial treatment effect in patients who were confirmed to have an ischemic stroke (5). Although the trial’s overall outcome was neutral due to the inclusion of many stroke mimics (patients with stroke-like symptoms who turned out not to have an actual stroke), its findings – along with an encouraging meta-analysis of early neuroprotection trials – suggest that ultra-early neuroprotective therapy could improve outcomes if the right patients are identified and treated quickly (5).

Thrombolytic therapy is also evolving. Accumulating evidence supports the use of tenecteplase (TNK) as an alternative to alteplase (tPA) for intravenous thrombolysis. Trials such as AcT and ATTEST have demonstrated that tenecteplase is at least non-inferior to alteplase in achieving good outcomes, with similar safety profiles, and some studies even show higher early recanalization rates with TNK (14, 15). Given its single bolus administration and ease of use, many stroke centers – and even entire regions (e.g., Alberta, Canada) – have transitioned to tenecteplase as the first-line thrombolytic. Ongoing trials are further investigating optimal dosing and specific subpopulations for TNK, but it is likely to become the new standard of care for lytic therapy in stroke. Several approaches are being evaluated to improve outcomes beyond macrovascular recanalization, especially to tackle downstream microvascular occlusions and the “no-reflow” phenomenon. One strategy is adjunct intra-arterial thrombolysis after thrombectomy. A small Phase II trial (CHOICE) initially suggested that administering a thrombolytic (alteplase) into the artery after a successful thrombectomy could improve final reperfusion and outcomes (16). However, two recent larger trials – POST-UK (using urokinase) and POST-TNK (using tenecteplase) – did not find a significant improvement in disability-free survival at 90 days with adjunct intra-arterial lytics (17, 18). In both POST-UK and POST-TNK, the proportion of patients achieving mRS 0–1 at 90 days was only slightly (and non-significantly) higher with adjunct intra-arterial therapy than with thrombectomy alone, tempering the initial enthusiasm generated by the smaller CHOICE study. More recently, however, the PEARL randomized clinical trial reported a statistically significant increase in excellent outcome (mRS 0–1 at 90 days: 44.8% vs. 30.2%; adjusted RR 1.45) with intra-arterial alteplase after successful reperfusion, without a clear excess in symptomatic intracranial hemorrhage (16). Taken together, these data suggest that while routine post-thrombectomy intra-arterial lysis cannot yet be recommended for all-comers, there is a biologically plausible and now trial-supported signal that selected patients with residual microvascular obstruction or fibrin emboli may benefit—keeping the concept of adjuvant IA lysis very much alive and shifting the focus toward refining imaging- and physiology-based selection criteria. Additional studies are underway, such as the multicenter TECNO trial evaluating intra-arterial tenecteplase for incomplete reperfusion. The stroke community is awaiting these results to see if refinements in patient selection or technique can make adjunct lytic therapy successful.

Importantly, macrovascular reperfusion (e.g., angiographic mTICI/eTICI) does not necessarily translate into tissue-level reperfusion. Several markers of microvascular reperfusion failure/no-reflow have been proposed, including incomplete parenchymal blush on angiography, persistent hypoperfusion on early post-EVT CT/MR perfusion, and discordance between vessel recanalization and downstream capillary flow. These markers are increasingly used to enrich trials testing adjunctive thrombolysis or antithrombotic strategies after EVT that aim to dissolve distal microthrombi and improve tissue reperfusion. NWU provides complementary information, because continued microvascular hypoperfusion can accelerate edema formation despite successful recanalization; consistent with this concept, perfusion-derived tissue-level collateral status (hypoperfusion intensity ratio) predicted post-treatment NWU and outcome in LVO patients (Faizy et al., J Cereb Blood Flow Metab 2021) (19).

Despite the major advances in acute stroke treatment, important gaps and uncertainties remain in treatment selection. For instance, patients with large-vessel occlusion but low NIHSS scores (i.e., relatively mild clinical deficits) present a dilemma – do they benefit from thrombectomy or not? These “mild stroke” LVO patients were not clearly addressed in the pivotal trials; some observational data suggest many do well without intervention, but others can deteriorate later (17, 18, 20). Randomized evidence in this group is still emerging, and trials such as MOSTE in France are ongoing, with inclusion nearly completed; until these results are available, it remains uncertain which low-NIHSS LVO patients derive clear benefit from thrombectomy. Again, the identification of patients at-risk for deterioration at baseline imaging is warranted in order to guide treatment decision-making.

Similarly, the role of thrombectomy for distal, medium vessel occlusions (DMVOs) has been tested in recent trials. The results of three randomized studies (DISTAL, ESCAPE-MeVO, and DISCOUNT) reported in 2025 were neutral to negative (9, 10). Adding endovascular therapy to best medical management for these smaller clots did not significantly improve 90-day functional outcomes versus medical therapy alone, and in some cases showed a trend toward higher risk (e.g., more intracerebral hemorrhages) with the intervention. These findings underscore that more intervention is not always better – and that careful selection of which patients truly benefit from aggressive treatments is critical. Overall, the emerging theme is that treatment selection matters. With multiple new therapies and expanded indications on the horizon, clinicians need better tools to identify the patients who will derive net benefit from a given intervention while avoiding futile or harmful treatments (21).

## The emerging role of NWU imaging in stroke

One promising approach to improving patient selection is the use of advanced imaging biomarkers, such as collateral imaging or quantitative net water uptake (NWU) (22, 23). NWU is a quantitative measure of early brain edema that can be calculated from standard noncontrast CT scans (24–26). Conceptually, NWU represents relative hypoattenuation of the early ischemic lesion. It is calculated by comparing the CT density of the ischemic lesion to that of normal brain (in essence, 1 – (lesion HU/normal HU)), effectively estimating how much water content has accumulated in the tissue. Higher NWU values indicate more severe vasogenic edema (greater water uptake), whereas lower NWU suggests less edema with higher probability of intact blood–brain barrier and chances of tissue salvage following blood flow restoration. Importantly, early NWU on the initial CT has shown strong correlations with stroke outcomes and complications across multiple studies (26, 27). Patients with a lower NWU (less edema) in the acute phase tend to have better functional recovery, whereas high NWU is associated with worse outcomes. For example, it has been found that among patients with large infarcts (low ASPECTS), those with low baseline NWU were much more likely to achieve good outcomes with thrombectomy, suggesting that NWU can stratify tissue viability beyond what is captured by conventional ASPECTS scoring (28–30). In other words, a large core on CT that has only modest water uptake might still contain salvageable (reversible) tissue, whereas a similarly sized core with very high NWU likely represents predominantly irreversibly damaged tissue. NWU thus adds a physiological dimension (edema severity) to the anatomical assessment of infarct size (31, 32). Within the broader imaging landscape, NWU should be viewed as complementary to, rather than a replacement for, CT perfusion, MR DWI/perfusion, collateral scoring, automated ASPECTS, NCCT radiomics, and deep-learning–based prediction of core and penumbra.

NWU has also been linked to specific risks like hemorrhagic transformation. Severe early edema reflects damage to the blood–brain barrier; studies have shown that regions with very high NWU on initial imaging are more prone to hemorrhage after reperfusion (33). In one analysis, patients with NWU greater than ~15% had a significantly higher incidence of symptomatic intracerebral hemorrhage post-thrombectomy. This suggests NWU might help identify those who need more careful blood pressure management or other precautions after reperfusion therapy. Similarly, NWU can improve prediction of malignant edema (massive swelling with midline shift requiring interventions like craniectomy). It has been shown that NWU outperformed conventional measures in forecasting malignant infarction: an NWU threshold of ~11% on early CT had a sensitivity of 97% and specificity of 98% for predicting malignant edema within 6 h of onset (27). Thus, NWU could alert clinicians to patients at high risk of life-threatening edema, prompting early aggressive management (for instance, intensive monitoring or timely surgical consideration) (34).

From a technical standpoint, NWU is appealing because it uses routine imaging (plain CT) without requiring contrast injection or specialized post-processing, making it in principle feasible to implement in most hospitals. It provides a continuos numeric value of tissue damage (water uptake), whereas standard NCCT assessment (e.g., ASPECTS rating) is more semi-quantitative. Automated software algorithms have been developed to calculate NWU rapidly, even incorporating it into ASPECTS regions for quick interpretation (the “ASPECTS-NWU” approach) (35, 36). In observational studies, NWU has been obtained in the emergency setting without delaying treatment, suggesting that it could offer additional insight in the critical early decision-making window, but its clinical use remains exploratory. By integrating NWU with existing criteria, clinicians may in future better distinguish which patients stand to benefit from therapies and which do not (37). For example, two patients might both have a large early infarct visible on CT; if one has a low NWU (suggesting that much of the tissue is not yet severely edematous and might still be salvageable), that patient might benefit from interventions like thrombectomy or edema-targeted drugs, whereas another patient with a similarly large infarct but very high NWU might be less likely to improve and more prone to complications no matter what interventions are done (29). At present, however, such scenarios should be regarded as hypothesis-generating, as no randomized trial has yet demonstrated a differential benefit of specific interventions according to NWU values.

Several limitations of NWU should be acknowledged. NWU estimates depend on scanner type, acquisition and reconstruction parameters, and local post-processing, which can introduce variability between centers. Thresholds associated with outcome or complication risk may differ across populations, time windows, and infarct locations, and standardized, widely adopted measurement protocols are not yet available. These issues need to be addressed through harmonization and prospective validation before NWU can be routinely incorporated into treatment decision-making.

Conceptually, NWU captures net water accumulation (early edema/BBB-related changes) and should not be interpreted as a direct surrogate of tissue viability on its own. Future studies could therefore combine NWU with emerging measures of tissue metabolism (e.g., oxygen extraction fraction/venous oxygenation mapping or PET/MR-based glucose metabolism) to refine tissue fate prediction and to better disentangle salvageable but edematous tissue from irreversibly infarcted tissue.

## Vasogenic edema in large-core stroke and implications for the core/penumbra paradigm

The classical core/penumbra model has served as the conceptual foundation of acute ischemic stroke imaging and reperfusion trials for more than two decades. In this framework, the infarct “core” is defined as irreversibly injured tissue, while the surrounding penumbra represents hypoperfused but potentially salvageable brain. However, accumulating evidence—particularly in patients with large baseline lesions—suggests that this dichotomous model may oversimplify the underlying pathophysiology.

A recent analysis highlights the central role of vasogenic edema and blood–brain barrier disruption in large-core ischemic stroke, proposing that early edema formation may fundamentally confound conventional imaging-based definitions of infarct core (38). In large infarcts, tissue hypoattenuation on noncontrast CT or severely reduced cerebral blood flow on perfusion imaging may not exclusively reflect irreversible cellular necrosis, but rather a composite signal driven by early water influx, endothelial injury, and microvascular dysfunction. As a result, imaging-defined “core” may represent a heterogeneous mixture of irreversibly infarcted tissue and severely injured—but still partially viable—brain. This concept is particularly relevant in the context of recent thrombectomy trials in large-core stroke. Studies relying predominantly on MRI-based selection have demonstrated benefit across a wide range of lesion sizes, whereas trials using noncontrast CT alone have yielded more neutral results (2, 4). One plausible explanation is that edema-related signal changes disproportionately inflate apparent infarct size on CT, especially in patients with rapid vasogenic edema formation, thereby obscuring meaningful biological differences between patients with similar ASPECTS but distinct tissue states (39–41). Net water uptake (NWU) provides a quantitative lens through which this limitation of the traditional core/penumbra paradigm can be addressed. By directly measuring the degree of tissue water accumulation, NWU captures an essential component of ischemic injury biology that represents a second dimension besides infarct volume (42, 43). In large-core scenarios, low NWU despite large lesion size may indicate delayed edema evolution and residual tissue resilience, whereas high NWU reflects advanced blood–brain barrier failure and a predominance of irreversible injury (44). From this perspective, NWU reframes the concept of “core” from a purely volumetric construct to a spectrum defined by edema severity and tissue vulnerability.

Importantly, this edema-centric view does not negate the core/penumbra concept but refines it. Rather than asking whether tissue is inside or outside the core, the critical question becomes whether the tissue has crossed a biological threshold beyond which reperfusion is unlikely to confer benefit and may instead increase harm. In this sense, vasogenic edema—and its quantification via NWU—may explain why patients with similarly large infarct volumes experience markedly different outcomes following reperfusion therapy.

Integrating edema-sensitive biomarkers into acute imaging workflows may therefore help reconcile discrepancies between trials, improve selection in large-core stroke, and move the field toward a more physiologically grounded, tissue-based treatment paradigm that extends beyond binary definitions of core and penumbra.

## Future outlook

Looking ahead, AI and deep learning will be the catalyst that makes NWU a rapid, routine vital sign of tissue state (19, 45). Purpose-built convolutional and transformer models can segment ischemic tissue and contralateral reference regions robustly, correct for artifacts, and output voxel-wise NWU with latencies measured in seconds—on the scanner console or Picture Archiving and Communication System (PACS)—so results arrive during door-to-CT, not after it. Crucially, DL enables prospective standardization across vendors and protocols by learning scanner-invariant features from multi-center data, while federated learning and continuous calibration curb site drift without moving raw images. Model outputs can include calibrated uncertainty and transparent overlays (sampled ROIs, HU deltas), allowing clinicians to audit why NWU is high or low at a glance and keeping a human-in-the-loop for edge cases (46). Recent prospective work with an explainable, non-DL pipeline already shows that NCCT-only automated NWU is feasible, accurate (≈1–1.5% MAE vs. CTP/DWI references), fast (<10 s), and generalizes externally—establishing a performance floor that DL can surpass on small/subtle lesions and noisy scans while preserving speed and reliability. Together, these advances point to a near-term reality where AI/DL delivers real-time, prospective NWU alongside automated ASPECTS, clot detection, and collateral surrogates, transforming NCCT into a tissue-profiling tool that informs selection without delaying reperfusion. Deploying this safely will hinge on guardrails—automated QC, versioned models with performance dashboards, and clear action frameworks—but the direction is clear: AI/DL is the bridge from technically possible NWU to universally available, seconds-scale NWU in everyday stroke care.

Beyond automated NWU measurement, AI-based collateral assessment is an active area of development. Promising CT-based approaches include deep learning models trained on single- or multiphase CTA (or dynamic CTA derived from CTP) that output an objective collateral score and quantify regional delay/contrast filling patterns; MRI-based options include time-resolved MRA and arterial spin labeling-derived collateral/perfusion features. Integrating automated collateral scores with NWU (and conventional perfusion metrics) could support reproducible, observer-independent patient stratification in large-core LVO and MeVO populations.

One important application of NWU imaging is in the context of neuroprotective and anti-edema therapies (13). As noted earlier, past neuroprotection trials may have failed in part due to treating a broad population where many patients were unlikely to respond (13). NWU could help identify a subset of patients who would be most likely to benefit from edema-targeting treatments. For instance, patients who already show high NWU (significant edema) very early might need aggressive anti-edema interventions (or conversely, they may be too far progressed for certain therapies), whereas those with moderate NWU could represent an optimal group for testing new neuroprotective drugs that aim to slow edema development. Researchers have been investigating agents such as Sur1-Trpm4 channel inhibitors, VEGF pathway modulators, and aquaporin-4 blockers (7, 44). If NWU can reliably measure the edematous process that these drugs target, it can be used to select patients (e.g., enroll only those who show early significant edema formation) and to monitor treatment effects. This approach – pairing a targeted therapy with an imaging biomarker of its target – embodies the concept of precision medicine in stroke. Ultimately, integrating NWU into clinical trials and practice could extend the therapeutic window and improve outcomes by personalizing intervention decisions based on the ongoing pathophysiology in the patient’s brain.

For neuroprotection trials that target mechanisms beyond classical anti-edema pathways (e.g., blood–brain barrier stabilization, anti-inflammatory strategies, mitochondrial protection, or microcirculatory modulators), NWU could be used as an early, quantitative imaging endpoint reflecting downstream edema biology and tissue injury severity. Practically, NWU may enable (i) enrichment of patients with low baseline NWU (slow edema progression) who have a longer therapeutic window for cytoprotective agents, and (ii) measurement of treatment effect as change in NWU (ΔNWU) on early follow-up imaging, alongside clinical outcomes.

In the setting of no-reflow/microvascular reperfusion failure, a combined imaging approach may be particularly informative: macro-recanalization can be documented angiographically, while residual microvascular hypoperfusion can be quantified on post-procedural perfusion imaging, and NWU can serve as a readout of the tissue-level consequence (edema progression). A trial framework could pre-specify a ‘no-reflow phenotype’ (successful recanalization plus persistent tissue hypoperfusion) and test whether adjunct strategies that improve microvascular reperfusion attenuate subsequent NWU increase and infarct growth.

In addition, acute ischemic stroke management will likely continue to evolve rapidly, guided by insights from these recent studies and emerging technologies. We can expect to see a greater emphasis on individualized care – selecting the right treatment for the right patient at the right time. Advanced imaging markers like NWU are poised to play a role in this personalization. Before NWU can be widely adopted, further research is needed to validate threshold values and ensure consistency across centers and imaging platforms. Ongoing studies are working to standardize NWU measurement methods and to seamlessly integrate NWU calculations into stroke workflows (for example, developing automated NWU tools that plug into hospital PACS imaging systems) (38, 42, 45). If successful, it’s conceivable that future stroke protocols will include automated NWU readings on initial CT scans, alerting physicians to edema severity in real time. Such information, combined with other clinical and imaging data (such as perfusion maps or collateral status), could refine decisions such as whether to attempt reperfusion in borderline cases, or how aggressively to manage blood pressure and swelling in a given patient.

Acute ischemic stroke care is increasingly moving beyond intravenous thrombolysis and/or mechanical thrombectomy alone toward combination approaches and adjunct treatments. Neuroprotective drugs, once considered a dead end, are being revisited with better trial designs and more targeted patient selection. The next generation of neuroprotection trials is already underway – for example, a new trial (ACT-42) is testing an updated nerinetide agent delivered ultra-early, building on the clues from FRONTIER and ESCAPE-NEXT about treating within the first 3 h (15, 35). There is cautious optimism that by treating patients within the first golden minutes of stroke (even en route to the hospital) and focusing on those most likely to benefit, we may finally demonstrate a tangible neuroprotective benefit. Likewise, research into optimizing reperfusion strategies continues – whether it is improving thrombolytics or enhancing mechanical techniques for different vessel sizes. The negative or neutral trials for distal occlusions and minor strokes do not mean these patients are doomed to go untreated; rather, they urge us to find novel ways to help them, perhaps through pharmacological approaches or intensive monitoring that target their specific pathophysiology (for instance, promoting collateral flow, preventing secondary injury, etc.).

In summary, the current state of ischemic stroke therapy is one of both solid progress and ongoing refinement. We have extended effective reperfusion therapy to patients with larger cores and simplified thrombolysis with tenecteplase. We have also recognized that benefit is not uniform across all subgroups, particularly in patients with very mild symptoms or very distal clots, where randomized evidence remains limited. The focus now is on sharpening our selection criteria – utilizing tools like NWU imaging and other biomarkers – to guide treatment decisions. By doing so, we aim to maximize the benefit–risk ratio for each patient, avoiding futile or harmful interventions while pursuing new treatments for those who need them. This personalized approach, supported by evidence from recent trials and innovative imaging, will shape the next chapter of acute stroke care. The hope is that in the near future, advances like NWU-guided therapy and ultra-early neuroprotection will join thrombectomy and thrombolysis as established pillars of stroke treatment, further reducing the toll of this devastating disease.

## Data Availability

The original contributions presented in the study are included in the article/supplementary material, further inquiries can be directed to the corresponding authors.
